# An integrative systems biology view of host-pathogen interactions: The regulation of immunity and homeostasis is concomitant, flexible, and smart

**DOI:** 10.3389/fimmu.2022.1061290

**Published:** 2023-01-24

**Authors:** Zvi Grossman, Andreas Meyerhans, Gennady Bocharov

**Affiliations:** ^1^ Sackler Faculty of Medicine, Tel Aviv University, Tel Aviv, Israel; ^2^ Vaccine Research Center, National Institute of Allergy and Infectious Diseases, National Institutes of Health (NIH), Bethesda, MD, United States; ^3^ Infection Biology Laboratory, Department of Medicine and Life Sciences, Universitat Pompeu Fabra, Barcelona, Spain; ^4^ ICREA, Barcelona, Spain; ^5^ Marchuk Institute of Numerical Mathematics, Russian Academy of Sciences, Moscow, Russia; ^6^ Institute of Computer Science and Mathematical Modeling, Sechenov First Moscow State Medical University, Moscow, Russia

**Keywords:** systems immunology, functional homeostasis, context discrimination, tuning, adaptive differentiation, cellular and cell population learning, smart surveillance, rinse and replace

## Abstract

The systemic bio-organization of humans and other mammals is essentially “preprogrammed”, and the basic interacting units, the cells, can be crudely mapped into discrete sets of developmental lineages and maturation states. Over several decades, however, and focusing on the immune system, we and others invoked evidence – now overwhelming – suggesting dynamic acquisition of cellular properties and functions, through tuning, re-networking, chromatin remodeling, and adaptive differentiation. The genetically encoded “algorithms” that govern the integration of signals and the computation of new states are not fully understood but are believed to be “smart”, designed to enable the cells and the system to discriminate meaningful perturbations from each other and from “noise”. Cellular sensory and response properties are shaped in part by recurring temporal patterns, or features, of the signaling environment. We compared this phenomenon to associative brain learning. We proposed that interactive cell learning is subject to selective pressures geared to performance, allowing the response of immune cells to injury or infection to be progressively coordinated with that of other cell types across tissues and organs. This in turn is comparable to supervised brain learning. Guided by feedback from both the tissue itself and the neural system, resident or recruited antigen-specific and innate immune cells can eradicate a pathogen while simultaneously sustaining functional homeostasis. As informative memories of immune responses are imprinted both systemically and within the targeted tissues, it is desirable to enhance tissue preparedness by incorporating attenuated-pathogen vaccines and informed choice of tissue-centered immunomodulators in vaccination schemes. Fortunately, much of the “training” that a living system requires to survive and function in the face of disturbances from outside or within is already incorporated into its design, so it does not need to deep-learn how to face a new challenge each time from scratch. Instead, the system learns from experience how to efficiently select a built-in strategy, or a combination of those, and can then use tuning to refine its organization and responses. Efforts to identify and therapeutically augment such strategies can take advantage of existing integrative modeling approaches. One recently explored strategy is boosting the flux of uninfected cells into and throughout an infected tissue to rinse and replace the infected cells.

## 1 Introduction

### 1.1 The overall perspective

The development of individuals from germ cells is programmed by evolution to attain precise functional organization, modular but dynamic, including sophisticated algorithms to allow dynamic adaptations to the ever-changing demands and conditions. The algorithms required to control perturbations by foreign agents are distinct from those that adaptively regulate other aspects of physiological variation but there is a substantial overlap. The nature of these latter algorithms is the central theme of this article collection and of this communication.

Cells manifest versatility of operational states and developmental options, not “plasticity” (1, 2). Cellular states are dynamically sustained or modified *via* interactions with other cells, especially those in their vicinity and those designed to participate in mediating local and central physiological control. At the same time, these reciprocal interactions dynamically tune the functional organization of groups of cells, of the tissue, and of the system. Contributing to reshaping the phenotypic and functional landscape are selective division, death, and migration events, which along with tuning result in premeditated quantitative and qualitative changes in tissue composition. Early fragmentary expositions of this perspective appeared in the literature long ago [e.g. (1–4)].

Systems Biology aims at producing global multilevel representations of the functioning body or parts of it in health and disease (5–7). Physiological organization of networks within networks is depicted in terms of the identifiable constituents (organs > tissues > cellular modules > cells > molecular modules > molecules). It is desired to simultaneously describe mathematically the dynamical interactions within and among subsets of the constituents embedded into spatial contexts, at different levels of organization, in order to simulate their stationary kinetic patterns and to explain and predict their responses to specific perturbations or demands, typically observed at a lower degree of resolution (e.g., a smaller subset of constituents; coarser spatial and temporal scales).

Each of the network’s elements, down to the intracellular modules, receives and produces a stream of biochemically encoded instructions. In response, the network’s parts manifest self-organization capacity. Given these fundamental capacities (rich communication and self-organization) and the power of natural selection, the biochemical networking rules have evolved to create a reliable algorithm for the development of new individuals from germ cells with high precision. The algorithms operating to adaptively maintain functional homeostasis in the completed organism are also enabled by the same global set of predetermined biochemical instructions, but as in the case of the organization of work in social insect colonies (8), to use a metaphor, these algorithms reflect a compromise between a predetermined division of labor among the modules and cells and flexibility in task allocation.

### 1.2 Focus on the immune system: historical overview, and where we stand today

Historically, the immune system has been excluded from the above outlined integrative “systemic view”. For several decades, influential immunologists, almost without exception, have theorized that peripheral lymphocyte populations are educated during their ontogeny phase to avoid consequential self-tissue recognition, and/or are designed to exclusively recognize and act upon the presence of pathogens *via* the inherent ability of the latter to trigger expression of pro-inflammatory molecules and “danger signals”. Cells belonging to the innate immune system such as NK cells were postulated to recognize aberrant cell-surface protein expression, to serve as major foreign antigen-presenting cells for lymphocytes (dendritic cells, macrophages), or play subsidiary roles. Mathematical modelers (“theoretical immunologists”) have mostly accepted the paradigm of the day. They usually described the time evolution of the overlaid interactions of a pathogen with host constituents – its cellular targets, and cells mediating the immune response – in classical population dynamics terms: a “game of numbers” essentially disconnected from other aspects of physiological regulation.

The limited relevance of such approach is increasingly appreciated [reviewed, (9)]. The immune system at large is now widely recognized as a tool evolutionary designed to assist in functional homeostasis, i.e., in enabling tissues and organs to operate properly. Eliminating pathogens and transformed cells is integral part of it, and self-recognition is general and essential. This is a relatively new development, although rooted in observations and theoretical work dating back several decades (see below).

Applied to immunology, systems biology extends the reductionist program, with a variety of complex, dynamically organized and dynamically coupled molecular modules and circuits as low-level building blocks (10–12). Modules, or motifs, are cellular control elements (13, 14): cell-surface associated receptors, protein networks operating at subcellular locations, and the chromatin, in its role as complex regulator of transcription (15). The modularity need not be entirely physical but rather related to the division of work: the modules are viewed as specialized molecular mini networks that respectively mediate and regulate the activation of specific subsets of network “nodes”: proteins mediating signaling cascades, RNA, genes, transcription factors, environmental factors, etc. With the onset of the COVID-19 pandemic, extensive efforts to apply network and control science methods to the host-pathogen interactions are ongoing [reviewed, e.g., (16). See also (17)].

But even in this modern attire of integrated nonlinear networks, using computational and dynamical-systems tools to explain multilevel phenomena, models of immune behavior tend to underestimate the versatility (“plasticity”) of cellular states and its significance for functional organization. One purpose of this communication is to help closing this gap. It appears that the following two general assumptions are implicitly made by most systems immunologists: (a) Immune responses, while highly complex, are essentially schematic and predictable. The functional properties of immune cells are mapped to a set of discrete and identifiable developmental lineages and states. Given such states, prototypical sets of receptor-matching molecular signals and their variations (inputs) define the cellular responses (the outputs). A unique high-dimensional mathematical function, analogous to a transfer function in engineering, theoretically models, in principle, the system’s output for each possible input. Redistribution of the cellular states may result, affecting the new inputs and outputs, and so on. Accordingly, the spatial and temporal patterns of organized immune responses are complex but, in principle, amenable to rigorous analysis and *in-silico* experimentation if the external signals and the internal sequences of instructions can be traced and the relations documented. However, insights into the coordination and orchestration of these multiple cellular responses are scarce. (b) It is the role of impacted tissue constituents, with the help of other reactive networks, to maintain or restore functional homeostasis, i.e., robust operational state, while the pathogen is removed or delimited or afterward. The capacity of doing this, with or without overt inflammation, is considered a systemic property called “disease tolerance”, or “resilience”, largely distinct from “resistance”, or “protection”, and mechanistically uncoupled from it (18, 19).

These conventional views are being revised. It was early observed that “cell function does not strictly correspond to cell lineage” (1). Evidence accumulates suggesting that nonlinear multilevel network models still fall short of reflecting the scope and profound nature of the integration that takes place at different levels and across levels; the very concept of distinct “levels” is ill-defined (20). Interdependent tenets that are gathering empirical support include (a) capacity of individual immune cells and cell populations to adaptively integrate information over time into their phenotypes and functional organization, comparable to associative and supervised learning by the nervous system (1, 9, 21–23); and (b) overlapping (and thus, integrated) functional roles of different types of cells, primarily lymphocytes and innate immune cells (spanning together the whole spectrum of leukocytes, including the growing family of innate lymphoid cells, ILCs) in maintaining or restoring tissue integrity and functional homeostasis in the face of infection or other insults (24, 25).

Thus, we and others theorized that individual cells dynamically modify or even acquire useful perceptive and effector properties that improve their context dependent performance. This is achieved through dynamic tuning of the signaling modules/motifs and subsequent chromatin modification and adaptive differentiation (9, 26–28). As a population, resident and recruited tissue cells were proposed to reshape their collective responses through feedback reinforced associative learning. Learning is guided not only *via* dynamic communication with the nervous system, which processes and returns information [e.g. (29)]. but also by locally generated cues of well-being and stress. The latter induce local stimulatory and suppressive effects. Together, local feedback and messages generated at the higher level supervise the selection and coordination of the cellular responses (1, 21, 22). Information processing taking place outside the tissue and locally are both instrumental in the progressive adaptation to changing conditions and demands. These propositions are in line with the “computational physiologist” perspectives offered by Denis Noble and colleagues (20, 30, 31).

### 1.3 Organization of this article

In the following several sections we discuss these evolving perspectives and their bearing on immuno-physiological responses in acute or chronic infection. Better insights into natural responses can improve the design of vaccines and other therapies. They also redefine the limits of mathematical modeling of core physiological processes (as opposed to modeling for heuristic, data mining and for data organization purposes) (9). The main messages of this communication are:

(a) The context dependent cellular acquisition of new phenotypes and functions is constrained “by design” but the actual gene expression profiles are highly variable and dynamic and reflect various characteristics of the signaling environment evaluated over time.
(b) The responses of immune cells to infection and to subsequent pathogen- and inflammation-related tissue injury are progressively coordinated across tissues and organs, and are attuned to physiological needs, through a process akin to brain learning.
(c) Despite these complexities, distinct modes of tissue-centered responses can be inferred from observation and further characterized in experimental and clinical settings *via* an iterative series of top-down and bottom-up investigations and mathematical modeling.

Section 2 overviews the key evidence-based theoretical developments that guided the new view of the immune system: a set of functionally integrated adaptive networks of cells that learn from experience in real time and perform smart surveillance of the tissues. Section 3 revisits the autoreactivity of lymphocytes and their role in classifying perturbations and updating their responsiveness through tuning. Section 4 highlights the peculiarities of the responses to acute versus chronic infection and their variable association with inflammation and tuning, reflecting the need for calculated, non-stereotypic responses when a straightforward resolution of the infection is unattainable or too harmful to tissues. The next four sections delineate a theory of cellular learning. Section 5 discusses what we mean by “learning” outside the brain. In section 6 we discuss, conceptually and semi-mechanistically, unsupervised immune learning of signal associations in peripheral tissues. In section 7 we cite evidence that associative learning is also a property of effector-memory cells post activation, with the important implication that tissue resident immune cells may actively tune their perceptive and outward signaling machinery to better communicate with other tissue cells. In section 8 we discuss supervised, feedback-reinforced learning, wherein environmental quality-control cues to the cells, generated locally and evaluated both locally and at higher levels, provide information regarding the collective performance of these cells in restoring functional homeostasis and influence the response. Section 9 explains why it is desirable to incorporate live-attenuated or inactivated pathogen vaccines in vaccination schemes and use appropriate routes of administration to enhance tissue preparedness. In section 10 we reason that the intricacies of information processing and dynamic adaptation would render comprehensive bottom-up simulation of the immune response to infections, and rigorous prediction of the outcome, a futile endeavor. Rather, section 11 recommends a deductive approach aimed at identification and then characterization of alternative strategies whereby the system deals with a pathogen and with restoring functional homeostasis; the connection to and utilization of current hypothesis-driven integrative modeling approaches are briefly discussed. A strategy that we have been focusing upon recently, “rinse and replace”, is revisited in section 12; and in section 13 we focus on SARS-CoV-2 infection as a current case study, citing intriguing preliminary evidence that appears to indicate implementation of this strategy in tandem with the classical immune response. A highly simplifying mathematical model, presented in **Supplementary Materials**, illustrates numerically how the strategy might work. The main points are reiterated in the Conclusion section.

## 2 Smart immunity revisited

The functional properties and physiological activities of cells of the adaptive and innate immune subsystems are overlapping, not distinct. Lymphocytes, NK cells, ILCs, neutrophiles, monocytes and macrophages, dendritic cells, and other leukocytes exchange overlapping sets of signals with resident tissue cells, with cells of the neural and endocrine systems, and among themselves (25). Activated autoreactive T cells that migrate to tissues may act as a new class of innate immunity cells (32), while ILC subsets functionally resemble certain subsets of lymphocytes.

First insights into the nature and purpose of these interactions were gained decades ago by realizing that lymphocytes are purposefully self-reactive (2, 33–37) and that they function in a manner that is profoundly context dependent and adjustable. There was a growing schism between the traditional concept of antigen-centered lymphocytes that function as narrowly specialized members of a defense system *vis-a-vis* their autoreactivity and the biochemical diversity and degeneracy of intercellular signaling and intracellular signal processing that was discovered. We proposed that lymphocytes “compute” a complex inner picture of their environment at all times, beyond their role of surveillance of pathogens, because they need to minimize and take care of peripheral damage and because they have general regulatory functions (33, 38, 39). We hypothesized that lymphocytes are involved in forcing and steering the turnover and differentiation of several types of cells (33). It was then conceivable that even outside the nervous system, cells are required to deal adaptively with rich classificatory challenges in perception of and controlling their environment (2).

A conceptual foundation for a theory of non-neural networks that learn from experience was laid (“towards a theory of adaptive networks”, within ref. (2), was followed by “contextual discrimination of antigens by the immune system: towards a unifying hypothesis” (1)). Several lines of evidence indicated that physiological messages to the immune system are encoded not only in the biochemical connections of signaling molecules to the cellular machinery of its members but also in the magnitude, kinetics, and time- and space-contingencies of sets of stimuli (2, 23, 26, 40–44). Individual cells were proposed to learn to respond preferentially to biochemical agents that connect to the cellular machinery when such agents were regularly encountered in the context of similar patterns – arrays of external signals evaluated over time. In parallel, the cells learn also to tolerate, within limits, quantitative fluctuations in the strength of interactions, by tuning metabolic setpoints and activation thresholds. Beyond limits, an abrupt change in strength may result in an overt response, entering cell cycle and/or involving qualitative changes in the cell’s gene expression, depending on its baseline characteristics and dynamic state. The tension between positive and negative adaptations of responsiveness results in enhanced context discrimination.

These initial insights have led to the development of concepts such as “dynamical tuning” and “smart surveillance” (reviewed, (9, 27)). Since these observations and conceptual framework pertain to all cells of the immune system and beyond (9, 24, 27, 45, 46), they rationalize the view of functionally integrated adaptive responses to pathogens and other stressors.

## 3 Perturbations and their classification by the cellular machinery

“What is the ‘signal’ to the immune system to use one mode of response or the other? The choice could be based, in part, on kinetic and statistical characteristics of the stimuli” (1).

Lymphocytes are organized into clones of cells that possess unique signal transducing receptors and are developmentally selected, positively and negatively, to bind each a set of self-antigens with moderate affinities. In addition to moderate affinity binding, calibration of the activation threshold of the receptor complex through dynamical tuning (also referred to in this context as desensitization or habituation) (26, 27), along with T regulatory cells (Tregs) (47), control this autoreactivity, preventing overt, destructive autoimmunity under normal conditions. The autoreactive repertoire of lymphocyte receptors is broad enough to generally permit further clonal selection by foreign antigens to occur when the latter are encountered in immunogenic setting: further selected are receptors that can specifically bind their “cognate” foreign antigens with high affinity.

However, as the qualification to “immunogenic setting” suggests, the immune system is not designed primarily to discriminate self from non-self. Rather, it is constantly engaged in classifying, responding to, and memorizing meaningful perturbations of the homeostatic states of its cellular and subcellular components (48, 49). Perturbation of a system is loosely defined as a transient change of state. Considerable effort has been invested in linking perturbations of lymphoid cells, especially lymphocytes and antigen presenting cells, to changes in the pattern of external signaling molecules. Perturbations are meaningful if they lead to a lasting functional change, reversible or permanent.

Within the cell, opposing positive and negative interactions dynamically maintain homeostatic equilibrium within and among the cellular modules. The temporally “incoherent” nature of these nonlinear interactions (50) renders them sensitive to the strength and other kinetic features of perturbations, allowing a module to operate as a biomolecular switch in classifying these perturbations and responding differentially.

## 4 Acute and chronic infection and the significance of subthreshold interactions

For lymphocytes, acute infection typically results in suprathreshold perturbations. Antigen binding and co-stimulation elicit kinetic competition between excitation and deexcitation factors (or, more generally, processes) that depends on certain quantitative characteristics of the stimuli, and the maximum resultant imbalance in the antigen receptor complex was proposed to determine whether an activation threshold is surpassed. The activation threshold in turn was postulated and then shown to arise from the presence of cooperativity (a positive feedback loop) among excitation products, which in association with the restraining force of deexcitation endows the receptor’s state with bimodular behavior (26, 48, 51) [reviewed, (27)]. It turned out that the proposed simple mathematical statement of the sensitivity-to-change hypothesis (26) was essentially akin to postulating an “incoherent loop,” a ubiquitous generic motif in biological networks (50).

Tunability of the activation threshold was also ascribed to the kinetic competition between excitation and deexcitation. It was reasoned that stimuli allowing deexcitation factors to keep up with the rise in excitation factors and block them, inhibiting receptor activation, transiently resulted in negative tuning (desensitization) of the receptor module, because of an inherently slower decay kinetics of deexcitation relative to excitation. Recurring stimuli of a similar magnitude, or that are slowly varying, would repeatedly update the level of tuning so as to maintain tolerance to normal variation. These quantitative rules do not necessarily hold for other intracellular modules.

Under the cover of activation-threshold tuning, subthreshold interaction with self-antigens in the presence of other signals regulates the tuning of other modules and other cellular properties. Such tuning can result in sensitization of signaling modules rather than desensitization. Thus, the ongoing integration of TCR-mediated signals and accessory signals in the interactive milieu could prepare lymphocytes to respond more efficiently, rather than less, upon activation by a cognate pathogen (26, 48, 52). Conversely, during strong, chronic antigenic exposure, negatively tuned CD8+ T cells adaptively differentiate and acquire an “exhausted” phenotype, preventing immunopathology (53). The tuning theory suggested that T cell exhaustion is an adaptation to chronic infection; accordingly, exhausted cells are not “anergic” but are capable of mediating alternative forms of immunity. Recently it has been shown that the state of restrained functionality of exhausted T cells during chronic infection is actively maintained by adaptive differentiation of a small population of precursors that express the transcription factor MYB and possess long-term proliferative potential, multipotency and repopulation capacity (54).

The basic theory has been corroborated and extended by the work of others (Reviewed, (9)). Wraith summarized advances made in his lab over the years in understanding the molecular signatures of adaptive tuning, particularly of CD4+ T cell responses, including during thymic selection, the immune response to chronic antigen exposure, and antigen-specific immunotherapy of autoimmune conditions (43). Antigen and checkpoint receptor engagement recalibrate T cell receptor signal strength. Consistent with the proposed hierarchy of adaptive events, subthreshold interactions induce tolerance to persistent stimulation through a limitation of T-cell receptor mediated signaling combined with epigenetic priming of tolerance associated genes. The recent article highlights many advances in our understanding of immune regulation that the authors trace back to the tuning hypothesis, including the development of antigen-specific immunotherapeutic approaches suitable for treatment of a wide range of autoimmune conditions (43).

In addition to a higher binding affinity of pathogen derivatives, another key factor that enhances the perturbation and receptor mediated signaling is pathogen sensing, mediated primarily by innate immune cells, which brings to the fore the inherent proinflammatory properties of bacteria and viruses (55). Together, these two factors allow stimulation to overcome the typically low or moderate level of baseline inhibitory tuning.

Empowered in these ways in the context of acute infection, pathogens often elicit time structured and self-limiting (56, 57) T and B cell responses involving receptor activation, clonal expansion, differentiation, migration, and a variety of anti-pathogenic effector activities. Tregs, in turn, inhibit pathogen sensing and activation or the events post activation. As the baseline concentration of Tregs is dynamically adjusted to a lower-level background of self-antigen driven, stochastic activation events, which they control, they act to enhance the selectivity of the immune response at the cell-population level (47, 58, 59).

However, if the “stereotypic” and time-limited endeavor to eliminate the pathogen fails, the inflammatory response evolves, ideally attaining the complexity and coordination among immune constituents required to achieve the original goal while restricting immunopathogenesis. This arguably requires real-time computation. The presumed aim is to strike a balance for the resolution of infection without detrimental inflammation.

## 5 Acquired cellular phenotypes and functions *via* adaptive integration of signals

“Given the complexity and unpredictability of the environmental contexts in which antigen is recognized, it would be advantageous for the immune system if lymphocytes could learn from the sets of stimuli to which they are exposed which response is required without the need for precise pre-programming” (1).

Ongoing perturbations train cells of both the adaptive and innate immune systems to anticipate patterns of environmental stress and properly participate not only in neutralizing pathogens but also in the maintenance of functional homeostasis (9, 24, 27, 45). Such training is akin to learning. Together, training and the subsequent execution of complex physiological functions have been termed “smart surveillance”.

As Gerald Edelman noted in 1978 (see (2), ref. 56), enormous epigenetic variation occurs in lymphocytes and hematopoietic cells as well as in brain cells, and much of this variation is probably related to subtle cognitive and functional differences and forms the basis for adaptive self-organization. (Epigenetic here means simply “non-genetic”.) The implication was that single cells have the computational capabilities for complex learning [see also references 226-231 in (60)]. It was proposed that cells of all types are not only programmed to respond to signals, that is, to “information”, but also participate in the definition of information while sensing each other’s activities (2). The favored mechanism was feature discovery by competitive tuning of intracellular modules. The goal is to categorize biochemical activity in the environment in terms of the most regular patterns and to generate, dynamically, a phenotypic mapping of those that can be used later (2). A contemporary article on biological learning, aiming at conceptually integrating cognitive science and systems biology, essentially rephrases (and greatly expands and elaborates) this concept: a cell (or a more complex biological “agent”) implements information processing that involves the construction of a representation, or internal model, of its environment (60).

While general interest in integrating cognitive science and systems biology is increasing (60–62), sporadic, longstanding efforts to conceptualize the immune system as a cognitive system [e.g., (2, 24, 37, 45)] and to tentatively define cell learning algorithms [e.g (1, 9, 21–23)] remain largely overlooked by mainstream immunologists (prompting Jeremy Gunawardena to make the not-entirely precise statement that “immunologists have not felt the urge, so far, to draw on the resources of cognitive science”). We now overview some of these efforts.

## 6 Single cell learning and immunity: explore, discern, adapt, and store

Tuning is key again. Viability of all types of immunocytes is actively sustained and their phenotype and function are dynamically shaped by the signaling environment through tuning of signaling modules and subsequent adaptive differentiation. Again, tuning represents short-term and reversible molecular memory of recent interactions. Adaptive differentiation converts a subset of these transient modifications into more stable forms of epigenetic memory. Tuning and adaptive differentiation generate and sustain the functional flexibility and heterogeneity that is instrumental as these cells perform nonclassical smart surveillance functions, including smart resistance to acute and chronic infections.

Individual immunocytes tune their “attention” to meaningful recurring patterns, or “features”, of external signals. They can acquire phenotypic memory of such features, which is initially transient and reversible but can eventually become stably imprinted and heritable. Early “proof of principle” of the induction of “cytokine memory” was provided by Paul and colleagues (63). Stabilization is equivalent to commitment, restricting the range of cell fates. Adaptive differentiation may be seen as fine-tuning (in the usual sense of disambiguation) of the familiar cell differentiation and maturation process, which proceeds along major preprogrammed pathways sequentially selected by specific molecular cues. “A committed cell needs only stimulation, for example by the T-cell receptor, rather than the full range of instructive signals, to reacquire the specific phenotype” (64). Similar to cytokine memory, homing and chemokine receptors “display individual and variable degrees of imprinting”, and “prolonged exposure to instructive signals appears to be crucial for the establishment of topographical memory” (64). Both transient and stabilized forms of cellular learning result in diversification of potential cellular responses to infection or other insults. They might also be beneficially manipulated.

Signaling patterns are reinforced or suppressed through dynamic interactions within and among the cells. A possible cellular mechanism to achieve selective responses to patterns of signals in the immune system would be based on inducible changes in the tunable signaling efficacy of coupled intracellular modules, in analogy with Hebb’s rule, proposed in neuroscience to explain associative, or “unsupervised,” learning (65). One early manifestation was “double Pavlovian conditioning”, taking place in parallel in the brain of experimental mice and at the level of the immune system, where the learned pattern included input from the neural system (23). We proposed that – in this example – lymphoid cells learned to associate signals originating in the central nervous system (CNS), delivered *via* neuroendocrine or autonomic nervous channels, with stimuli by antigens and other immuno-active agents, in a way that leads to storage of this association. In the immune system, associative learning would be linked to intrinsic cellular tunability, not limited to receptor expression (23). Obviously, temporal association as such is ill-defined and is only one of several characteristics defining “features” that cells require in order to generate a useful phenotypic mapping, or representation, of their environment (2, 60). [For a useful glossary of terms from learning theory, such as associative memory, see reference (66)]

A conceptual model of unsupervised cellular learning was delineated (1, 9). The T cell receptor module is linked horizontally to other membrane associated modules, which receive different signals *via* their respective receptors, and vertically to downstream modules. So are the other membrane associated modules. The intracellular modules interact with each other and transduce signals among themselves and downstream, eventually targeting/recruiting transcription factors as well as effector proteins that influence the dynamic state of the chromatin and thereby impact the gene expression profile. The interactions among modules result in reciprocal stimulation of some, which in turn leads to the suppression of others – presumably because of attendant, cross-reactive, negative feedback – depending on their current states of tuning and on the patterns received. It can be readily envisioned, though still mechanistically poorly understood, that recurring patterned stimuli to the cell enhance the connections of a subset of modules to each other while suppressing the other connections. At this point, external signaling patterns may be divided into those that are mostly orthogonal to the learned pattern, in some “cooperativity space”, and those that are essentially parallel.

Thus, in analogy with the theory by Bienenstock, Cooper and Munro of cortical synapse modification (67), a sliding “modification threshold” was envisioned, where the “weights” of signals are adjusted through ongoing stimulatory experience, separating signal associations that are increasingly recognized and transduced from those that are increasingly suppressed, with convergence onto the dominant feature (1, 9). Such selective recognition of a signal association results in the sensitization of the signal transduction machinery to signals that occur as part of a particular extracellular pattern (by influencing post-transcriptional networking) and in the priming of a specific gene expression signature.

Our core hypothesis of experience based, Hebbian-like associative learning by single, non-neural cells has been recently restated (61). This notion is of sufficient importance to bear repetition and expansion, particularly in view of advances in the understanding of the biochemical modes of information acquisition and storage. In addition to positive and negative tuning (sensitization and habituation), intracellular module rewiring (modified signaling cascades), and adaptive differentiation (chromatin and transcriptional memory), changes in protein level, protein localization, protein activity, and protein–protein interactions allow cells to vary the sensitivity, duration, and dynamics of the response (68).

When analyzing the processing of high dimensional inputs by cells of the immune system recent studies focus on several “networking” issues: on the hierarchical organization of the intracellular modules and their (nonlinear) interactions in archetypical cell types (13, 69); on the heterogeneity and environmental context dependency (“plasticity”) of the differentiation and cross-differentiation of such cells, which appear to challenge the classic depiction of differentiation in terms of a branching lineages tree (70–72); on discerning qualitative characteristics of the input patterns from quantitative aspects (13, 44, 72, 73); and on revealing multiple antagonistic excitation-deexcitation loops that mediate threshold-dependent activation and tuning of signaling modules downstream the antigen receptor (in CD4 and CD8 T cells and in B cells) and thereby enhance selectivity in the expression of effector functions in response to perturbations, balancing tolerance and immunity (74–76).

These are important issues, but we are still far from a mechanistic understanding of the learning algorithms, which link incremental adaptation (e.g., from positive and/or negative reinforcement) to smart immunity: how temporarily patterned inputs are transformed into distinct, memorized cellular responses, how reversibly tuned states become stably differentiated, and how immune cells utilize these processes, learning to coordinate their responses so as to neutralize pathogens while promoting the functional integrity of tissues.

We will further discuss perspectives of immune learning, and the implications, after first considering some additional evidence supporting the feasibility of such learning in the tissues.

## 7 Effector memory T cells learn as well

For lymphocytes, the receptor for antigen is the primary signal transduction device. Dynamic tuning was originally introduced as a mechanism for reversible adjustments that take place mainly during subthreshold antigenic stimulation events of naïve and central memory cells. It was postulated to form durable phenotypic and functional diversity through subsequent adaptive differentiation. These mechanisms are responsible for subtle commitment events during ontogeny (e.g., in thymus), accompanying major lineage differentiation, and in the peripheral organs and tissues.

As cells mature, they gradually lose both self-renewal capacity and pluripotency. Yet, recent studies demonstrated that even the progressive differentiation of lymphocytes into effector memory cells following activation and clonal expansion is adaptive in nature [reviewed, (77–80)]. While acute infection elicits a relatively stereotypic sequence of division and maturation events, the actual biochemical pathways that determine gene expression patterns are varied, depending not only on the developmental states of the responding lymphocytes and on their tuning states and acquired features – that is, in our terms, on their previous experience in response to subthreshold perturbations (26, 52, 81), – but also on post-activation experience. Dynamic variation of signaling pathways and chromatin modification orchestrate the establishment and maintenance of distinct states of T-cell fate determination and functional commitment, including metabolic, localization, and trafficking properties (78, 80). This has implications for a better understanding of the diverse consequences of acute infections and for enhancing vaccination schemes. The cited studies highlight also regulatory networks and chromatin changes that contribute to maintain T-cell identity once established and impede the reprogramming of specific T-cell states.

Explaining how the immune system adjusts its response to the environment in which antigen is recognized remains a challenge. Citing (77) and references therein, “the composition and function of the immune cells need to continuously adapt to the environmental stimuli to preserve its responsiveness and protect the host (26, 82). This adaptation is not based on a germline heritage, but rather on the acquisition and inheritance by the T-cell clone progenies of epigenetic modifications and transcriptional changes following antigen exposure (83). …The nature, the dose of the pathogenic antigen, and the environmental signals dictate the magnitude of the T-cell responses, the acquisition of effector and inflammatory functional properties by activated naïve cells followed by the establishment and maintenance of memory cells (84, 85)”.

Adopting a broad perspective of an integrated smart surveillance system in protection and in maintaining functional homeostasis (9, 18, 24, 45, 81), it may be proposed that learning signaling features is also part of the physiological training (86–88) that innate immunity cells undergo in tissues such as skin and lungs (89). Since these cells generally lack a widespread major signal-transduction receptor, whose threshold dependent activation results in proliferation and/or differentiation and/or overt expression of effector functions, as do lymphocytes, an operational definition of subthreshold interactions here would be those that, short of overt activation, induce modifications at the intracellular signaling, transcriptional, and epigenomic levels that render the cell resistant to “noise” but “poised” for rapid transcription and expression upon stronger stimulation by signals associated with the training pattern. Subcellular sites of innate immune signaling (signaling organelles called “supramolecular organizing centers”) ensure digital cellular responses in a context dependent manner (90), as is the case for B and T cells. Some transiently activated cells may return to a resting state, with new changes imprinted depending on context. In this way, antigen non-specific priming of immune cells generates a memory-like phenotype (28, 87, 89). Both myeloid and lymphoid innate immune cells alter their responsiveness to stimuli through epigenetic reprogramming. The idea that innate immunity cells are tunable, undergoing silent training, or education, as they mature and differentiate, is an old one (91). We also predicted back then education of NK-like cells to acquire various helper functions; such cells have recently been characterized and form a subset of ILCs. A lot is also known at present, though much remains to be learned, about the receptor proximal signaling events, histone modifications, and metabolic changes occurring within individually trained cells in the presence of pathogens (90). A major challenge is deciphering, conceptually and mechanistically, how these events are orchestrated as these cells participate in a coordinated systemic response.

## 8 Supervised learning and immunity: explore with quality control, select, store, and retrieve

At higher levels of organization, the ability to learn and trace recurrent patterns may be utilized in intercellular communication to coordinate and optimize physiological functions and responses. There are two modes of learning: (a) “developmental”, whereby the cells follow predetermined or *ad-hoc* environmental cues; (b) supervised, or “reinforced”, in which the environmental cues to each cell are provided by other cells in the vicinity and include also feedback messages that are geared to the collective performance of these cells. The latter are generated locally and evaluated both locally and at a higher level, notably the brain (1, 21, 22). Inflammation is the setting of such events, whereby destructive immune responses aimed to eliminate pathogens are to be balanced against minimization of peripheral damage. Inflammation is expression of the coordinated response to infection, stress, and malfunction. Whereas unsupervised correlation learning reinforces correlations that are frequent, supervised correlation learning reinforces correlations that are good (66).

The underlying learning process can be divided into phases. First, exploration. We discussed earlier how recurring patterned stimuli to a cell may enhance the connections of a subset of modules to each other while suppressing other connections. A conceptually similar mechanism may apply at the cell population level. The interactions among cells may result in generalized reciprocity, such that within a given subset all members of the subset simultaneously adapt to receive and return complementary patterned stimuli. Subsets may compete as do cellular modules, which would result in a spontaneous selection of a subset in an unsupervised manner.

Selection overlaps and follows the exploration phase in the context of inflammation. The adaptive responses of individual cells discussed earlier are coupled to the nonlinear dynamics of diverse stimulatory and suppressive interactions operating at the cell-population and systemic levels. Here, interconnected network cells simultaneously teach and instruct each other, although such learning is developmentally constrained. Together such multilevel interactions provide rich opportunities, when the network is confronted with infectious pathogens or other stressors, for the selection of alternative forms of coordinated cellular activities, or responses. Selection may be guided dynamically by feedback from the tissue, e.g., *via* short term stress signals that vary in space and time. Those feedback signals bias the selection of functionally predominant subsets. The theory does not require specific feedback signals for each cell. Rather, fluctuations in one or more feedback signals, reflecting changes in the collective performance of the system of cells and enhancing or suppressing cellular activity in a quantitative manner, can result in a convergence towards the desired reorganization (92). If the signal is a local measure of “stress” in the tissue, it is reinforcing an organization that becomes increasingly correlated to reduction in the stress. The analogy with brain learning is clear: “incremental adaptation from positive and/or negative reinforcement explains how [a system] can acquire knowledge from past experience and use it to direct future behaviors toward favorable outcomes” (66). Interestingly, cells in various tissues secrete serotonin in the setting of damage or infection, and this factor, known as a modulator of neuro-activity involved in brain learning, can bind to different immune cells that bear receptors for serotonin and thereby fine-regulate their sensitivity to other signals (93).

The epigenetic memory of a transiently acquired organization is stored. The individual cells are primed such that the transcriptional machinery is poised for expressing the pre-selected sets of genes when reactivated. Finally, the collective information is dynamically retrieved. The regrouping of reciprocally signaling immune cells and infected tissue cells is faster than in the previous challenge or training episode. Importantly, the process is “associative” in nature, at the higher level, as is the recoupling of signaling modules in a single cell, requiring the activating patterns to be similar or partially overlapping, but not necessarily identical, to elicit a similar type of response. “Information acquired across different episodes or time points can be linked, thus offering an opportunity to … make novel predictions about the environment”. This was said in the context of “higher-order conditioning” in neuroscience (94), but the core principles are not that different. The importance is that a trained organ or tissue can infer the significance of present events by reference to those experienced in the past and adapt more efficiently based on this information.

Such feedback-reinforced learning during infection-induced inflammation would facilitate (a) readjustment of ongoing innate and antigen-specific responses, including both gross class selection and fine modulation; and (b) beneficial participation of recruited and tissue-resident lymphocytes and non-antigen-specific effector cells in the repair and healing process. Indeed, “immune cells change rapidly and subsequently produce factors that are required for the repair of tissue damage” (95). The antigen-specific and tissue maintenance functions are coupled and simultaneous, not inherently distinct.

## 9 Integrative view of tissue physiology and immunity: some implications of an emerging paradigm

A compelling case for a tissue-centered approach to natural immunity and to vaccination against infection has been made in a recent review on tissue immunity to SARS-CoV-2 by Donna Farber and colleagues, based on studies from her lab and others (96). The need to establish tissue resident memory T and B cells with the right composition, broad reactivity, and sustainability was emphasized. Those lymphocytes were phenotypically tissue-specific and discernable from their circulating counterparts. It was noted that coordinated processes between the innate and adaptive immune systems are essential to neutralize infections with minimal damage to the host, although the notion of adaptive acquisition of functional phenotypes locally by innate and virus-specific immune cells alike did not come up.

As discussed, we and others view the infected tissue, including resident immune cells and neuroendocrine cells, as the core of an integrated, adaptive, information-processing and information-storing system. This “learning” perspective adds a theoretical dimension to other observations that are shifting research interest towards tissue-centered immunity.

Consider for example the case of vaccination against SARS-CoV-2 infection. Several studies have indicated that SARS-CoV-2 breakthrough infection in previously vaccinated persons, or vaccination of previously infected persons, induces more robust immunity – so-called “hybrid immunity” – than mRNA-based vaccination and boosting alone [e.g., (97)]. T cells are considered the strongest immune correlate for vaccinated and convalescent individuals avoiding hospitalization (98), and both the broader specificity of these cells against the virus associated with hybrid immunity and the priming of targeted tissues such as the upper and lower airways may account for enhanced protection [e.g., (99)].

From a learning perspective, since cellular memories of response to pathogens are imprinted both systemically and in the targeted tissues, it is desirable to incorporate live-attenuated or inactivated pathogens in vaccination schemes and use appropriate routes of administration to enhance tissue preparedness. Preexisting responses to vaccines may condition the tissue to mediate early control. The immunity to be attained would resemble hybrid immunity, while sparing the part of experiencing a risky infection. Furthermore, intuitively, the closer the non-natural challenge is to the anticipated one, the higher the quality of tissue and systemic preparedness. Therefore, a live-attenuated virus vaccine is expected to be superior to inactivated vaccine, though both provide stimulation at the normal infection site. Administrating attenuated and mRNA-based vaccines simultaneously would combine the advantages of both, while directing *all* immune cells to the relevant sites of inflammation. Safety issues, related to a potential adverse reaction of some people to the deliberate triggering of inflammation, would need to be resolved in advance.

In general, the stress to the tissue exerted by the training challenge is not and need not be quantitatively comparable to that caused by a pathogen to promote a qualitatively similar corrective inflammatory response. This is supported by two kinds of theoretical considerations. The first is the generalized associative nature of training (Section 8), which is analogous to, and generalizes, the structural cross-reactivity of immunological memory generated by conventional priming of the adaptive (pathogen specific) immune response. Tissue-centered vaccination triggers a coordinated inflammatory response to the immunizing agent involving tissue-resident and recruited immune cells as well as authentic tissue cells (including neurons) that are directly or indirectly perturbed by that agent. Because the stability properties of steady states in a dynamical system, the tissue in this case, limit the dependence of the outcome on initial conditions – reflecting the flexibility of the forces that hold the nested cellular and molecular networks together – the perturbation induced by the vaccine needs only to resemble, or partially overlap, a real challenge to provide useful training. The adaptive intracellular modifications and intercellular rewiring associated with the response are stored, facilitating a rapid recall upon challenge with the live pathogen, though further tuning may then be required. All this would happen in tandem with the systemic buildup and recall of a conventional antigen specific memory.

The second, complementary argument implicit in this scenario is based on the expectation that much of the “training” that a living system requires to survive and function in the face of disturbances from within and outside is already incorporated into its design, so it does not need to deep-learn how to face a new challenge each time from scratch. Deep learning is successfully implemented in artificial-intelligence applications but requires vast numbers of training examples. Instead, living systems often learn from experience how to efficiently select a built-in strategy, or a combination of those, and then require parameter tuning to refine their organization and responses.

Potential built-in strategies are further discussed in the following sections. Those are supposed to have been learned by evolution; a strong analogy has been drawn between reinforced learning as it operates in an individual – reusing behaviors that have been successful in the past – and the way selection increases the proportion of fit phenotypes in a population (66). The existence of a limited set of evolutionary learned tissue-response strategies means that tissue cells and tissue-seeking immune cells may be already poised, or prepared, to form or strengthen interconnections that realize a chosen strategy, in response to stimuli that resemble a perturbing pattern genetically stored in their collective memory. Training *via* an agent that evokes a similar pattern may boost the recall process, as described. Thus, connections engaged during vaccination-induced inflammation and the resulting cellular modifications may often correctly predict the nature of the required response to a future challenge by the live pathogen. In that case, the numerical and phenotypic modifications of the immune system stored during tissue-centered vaccination are anticipatory of infection both quantitatively (number of memory cells) and qualitatively (immune cells poised for tissue-sparing responses), enhancing preparedness. This suggestion is testable: it might be possible to verify, clinically or in animal experiments, that such vaccination promotes, by association, selection of the right protective strategy.

## 10 The details of the integrative response to infection are inherently unpredictable

Unless the pathogen is rapidly overwhelmed by an overshooting wave of “stereotypical” effector cells, the *ad-hoc* nature of information processing and ongoing computation in and around an infected tissue generally precludes precise mathematical simulation of the immuno-physiological responses to acute or chronic infections – not only in practice, due to a limited understanding of the system’s constituents as multilevel networks, but even theoretically.

First, during lingering infection, the system is typically far from equilibrium by several measures. Second, the cellular network to be modeled is always a subsystem within the body, so it is practically an open system, dynamically exchanging signaling and effector molecules with organs and tissues that are not accessible to micro-modeling (the central and autonomic nervous systems, the neuroendocrine complex), and recruiting from hematopoietic and lymphoid tissues a heterogeneous population of immunocytes whose (varying) composition and functional repertoire is unknown and idiosyncratic (100, 101); in this scenario, recruited and genuine tissue cells transiently merge into a new network entity in a manner that is virtually unpredictable. Third, the profound context dependency of cellular responses to biochemical stimuli is linked to the complex functional operation of individual intracellular modules; for example, in the roles of histone-modifying enzymes and their associated chromatin modifications in transcriptional regulation. Thought earlier to represent a hardwired deterministic code, histone modifications have more recently been found to function in a cellular-context-dependent manner with multiple potential outcomes depending on various factors, including the relative concentrations of downstream effector proteins (102). Fourth, the multifactorial response properties of each element of the network, down to individual cells and molecular modules, are adaptive, shaped by experience over time. The adaptation is feedback reinforced (“supervised”) *in-situ*, postulated to dynamically couple immune defense to tissue homeostasis.

Together, such intricate dynamics would render comprehensive bottom-up simulation of the response, and predicting the outcome, a virtually impossible challenge. Therefore, even if the biochemical interactions that normally help the constituents to work together in harmony were well understood, in some averaged sense, top-down analysis and phenomenological modeling – involving observing, guessing, and consistency testing – are indispensable in order to identify response strategies and to associate them with cellular and multicellular mechanisms, structures, and relevant biomarkers.

## 11 Prototypic strategies of response can be inferred using deductive reasoning

Adopting a somewhat optimistic outlook, it may be considered that the enormous complexity of the biomolecular networks and embedded control elements within cells, and of the inter cellular communication mechanisms, which also couple different levels of organization to each other, are “designed chiefly for the purpose of enabling reliable cell [and cell-assembly] decisions concerning relatively simple behavioral functions in uncertain and variable environments” (14).

The assumption of prior existence of a limited set of tissue-specific core strategies raises the prospect of identifying those through observation and deductive reasoning. While a detailed realistic description of the response cannot be derived from a multilevel mapping of constituents and biochemical interactions *per se*, extensive as it may be, the hope that a nominally high-dimensional biological system may effectively behave as a relatively low-dimensional dynamical system is not farfetched: several examples support the notion that there are simple organizing principles that allow a lower-dimensional representation, but only if prior knowledge about these principles exists. Conceptual understanding is a prerequisite. The commonly advertised notion that new biological properties can be “revealed” through, or “emerge” from, analysis of nonlinear models that faithfully reconstruct limited sets of data is also a delusion (9). When prior phenomenological understanding does exist, the main value of a mathematical representation is heuristic; it does not “predict” novel outcomes and behaviors, but it could help us interpret empirical observations, identify relevant biomarkers and kinetic mechanisms, and rationalize research directions or clinical approaches.

Thus, an alternative to an inductive, bottom-up modeling, from the specific biochemical processes to the outcome, is using broad observations to abstract the existence of specific alternative strategies whereby the system deals with a pathogen and with restoring functional homeostasis and focusing on those strategies. This would allow inferences to be made regarding the significance of specific events and biomarkers in the execution of such a strategy. Instead of models that explain in detail “how the system works”, we would have explanations of “what it is doing” at a coarser level, allowing us to identify early correlates of effectiveness and failure. Enhancing the underlying “good” mechanisms *via* tissue targeted vaccination (and potentially adjuvants and other treatments to recruit key players) would be the most promising way of using such knowledge in the clinical setting.

Inferring the rules governing a dynamical process from the observable patterns that it generates is a fundamental scientific issue (103). Here, a major challenge to researchers is to identify additional strategical components of protection that are regularly manifested in response to infection and that are associated with favorable outcomes even in patients with risk characteristics. Lacking *a priori* knowledge, a novel strategy is hardly expected to be discovered by means of a formal mathematical or statistical exploration of the data alone; instead, the human gift for abstraction and generalization can be helpful.

Yet, extensive sampling of affected tissues, phenotypic characterization and transcription profiling, and statistical exploration of the data can greatly assist with the discovery process. They reveal site-specific immune dynamics that are associated with protection or immunopathology but are not readily observed in circulation (96). We may expect distinct patterns in different classes of patients and vaccinees and at different stages of infection and disease. In particular, comparing transcriptome information from immune cells present in the circulation and in samples from accessible tissue extracts under these different circumstances may reveal (a) the signatures of tissue specificity in lymphoid and non-lymphoid immune cells, and (b) the coactivation signatures presumably associated with different strategies of protection; correlation with improved disease outcome may guide efforts to understand the nature of those.

Once a strategy is inferred from observation, its implementation may be studied using a variety of methods. Focused observations in experimental non-human models or in an identified subset of patients can be designed to follow the course of putative cellular events and changes in gene expression profiles. For example, imaging can be used to develop a better understanding of how the innate and adaptive immune systems carry out the task: dynamic intravital imaging can reveal basic information about cell migration and interactions within lymphoid and non-lymphoid tissues, while emerging high content static imaging methods reveal in great detail the types and states of immune and non-immune cells in tissues (104). A minimal set of predictive variables can be extracted from a larger hypothetical set, embedded in a large database, by procedures that involve pattern recognition and dimensionality reduction (103) (for a recent example in a different context, see (73)). Minimal mathematical models can be formulated, once the variables are specified, and the parameters estimated.

An insightful study, rather unique in its choice of a deductive, top-down approach, has analyzed the immune response to malaria infections (105). The response was broken up into components with distinct effects on cell birth and death, quantifying the impact of each on disease and pathogens. It demonstrated that hosts control infections not only by killing pathogens, but also by starving parasites and shortening the lifespans of cells on which they depend. The analysis suggests that these strategies “are deployed in a coordinated fashion to realize distinct resource-directed defense strategies that complement the killing of parasitized cells” (105).

A conceptually simpler, but methodologically similar top-down modeling approach has recently been adopted in relation to SARS-CoV-2 infection. The importance of rapid recruitment of specific B and T cell responses to the virus and implementing innate modes of resistance have been suggested by many studies and can be viewed as the “basic strategy”. Less is known about the coordination and interdependence of these mechanisms; a testable assumption is that they need to be coordinated both in time and scale. Grebennikov et al. recently developed a calibrated mathematical model of antiviral innate and adaptive immune responses to SARS-CoV-2 during mild-to-moderate severity infection (106). Using published data from human challenge studies and other available data and estimates, they inferred the sensitivity of the peak viral load to the kinetics of the responses. Because the model does not describe the full range of functions ascribed to the immune system in preserving and restoring tissue integrity and functional homeostasis, it cannot be expected to be fully explanatory or predictive. Yet, interpolating from the training data to other points in the same general domain, using a well-calibrated model, is known to possess considerable statistical validity.

We next draw attention in some detail to a novel anti-viral strategy: boosting the flux of the targeted cell population by inducing the proliferation and differentiating of uninfected precursors, to rinse and replace infected cells without directly eliminating them or blocking their infection.

## 12 Rinse-and-replace: a natural mechanism of homeostasis and defense

**Figure 1:**
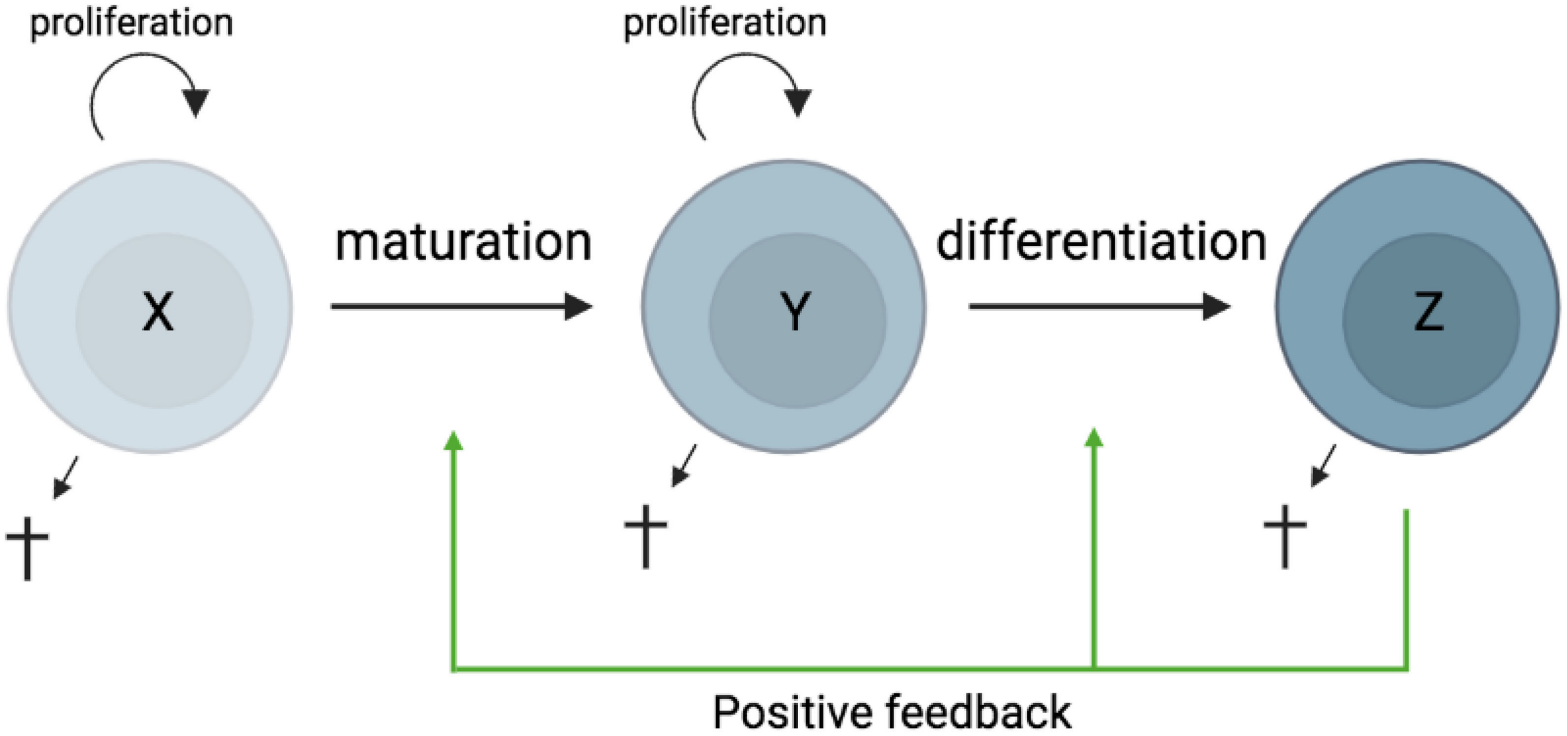
Schematic presentation of a simple feedback-controlled balance-of-growth-and-differentiation model. X, progenitor cells; Y, maturing cells; Z, terminally differentiated cells.

In most tissues, cells are constantly generated and removed through division, differentiation, migration, and death in an orderly manner. Flux is the process whereby cell populations of different maturation stages progressively replace each other while the overall phenotypic profile is (largely) maintained. Feedback mechanisms control the balance between renewal and differentiation of progenitor cells (56, 107–110) [reviewed, (9)]. **Figure 1** is a schematic depiction of a simple feedback-controlled balance-of-growth-and-differentiation model.

Note that Y cells in **Figure 1** generally represent a heterogeneous population, with subpopulations generated from their immediate progenitors in a series of feedback-controlled maturation steps that may involve context-dependent splitting into distinct differentiation pathways. On average, differentiation is associated with maturation.

A basic assumption of this model is that the progenitors are less susceptible to feedback differentiation pressures than their progeny, and this can impart “stemness” to the progenitor compartment while the progeny, despite possessing self-renewal capacity, must assume transitory kinetics. Irrespective of mechanistic details, relatively differentiated tissue cells are dynamically rinsed and replaced by their progenitors, at a rate that can be physiologically controlled, and this has been considered a natural control mechanism in health and disease [reviewed, (9)].

The model pertains, for example, to the concomitant regulation of immune activation and homeostasis (110). It implies that, under recurrent clonal (or polyclonal) T-cell activation, the activated population must be in flux: extensively proliferating memory phenotype T cells subject to feedback-mediated differentiation pressure are progressively pushed forward and out, along their preprogrammed or *ad-hoc* developmental pathways, being replaced by the progeny of activated naïve cells. The number of naïve cells in turn is maintained dynamically also *via* regulated incorporation of recent thymic emigrants. Independent of the precise mechanisms of feedback control, there is a sound physiologic rationale for a dynamic flux, in the context of recurring inflammation and activation. Constant cell replacement acts to reduce the accumulation of detrimental (e.g., tumorigenic) mutations associated with repeated episodes of extensive proliferation; and it also confers “functional resilience,” flexibility in readjusting the composition of effector cells to varying physiologic needs (110).

As mentioned above, it is a long-standing proposition that boosting the physiological turnover of populations containing malignant or otherwise deficient cells, rather than targeting these cells directly, might be commonly adopted by the immune system as a preferable strategy. In the context of tumorigenesis, this was termed “immune surveillance without immunogenicity” (33). Indeed, targeting transformed cells directly is problematic because tumor-associated antigens are often not immunogenic. Moreover, as long as transformed cells maintain a degree of functional integrity, the information from the tissue as processed by the system’s cognitive quality-control apparatus may be interpreted as requiring inclusive protection from attackers from within; from this perspective, evading immune surveillance may be partly attributed to misguided physiological adaptation rather than to adaptation of the tumor cells. On the other hand, implementation of the accelerated flux strategy requires only sensing that there are “misbehaved cells” present, and those would be washed out along with their normal counterparts and replaced. If the transformed cells are still partly responsive to differentiation-inducing signals, and provided also that the hierarchy of resistance to such signals is not reversed in the tissue, so that normal progenitor/stem-cell like cells exist that can still enforce transitory kinetics on the transformed cells – then progression of the tumorigenic process can be curtailed (111). There is evidence that resident T cells and innate immune cells may indeed assist in tissue differentiation and development, and that disruption of such activities may result in tumorigenesis (112).

In untreated HIV infection, regular activation of intrinsically long-lived provirus-containing (“latently infected”) CD4+ T cells, rather than continuous chains of virus production and infection, is responsible for sustained infection (113). Immune activation also increases the turnover of these cells (113). Provirus-containing cells are washed out as a result of their spontaneous or antigen induced activation, which when coupled to viral protein expression can induce local inflammation and recruitment of uninfected cells to the activation sites, boosting a feedback-controlled flux and causing latently infected cells to be progressively rinsed and replaced. *De novo* infection counters this washout, but the boosted flux limits the viral load, resulting in a long-term natural control (113). Note that since expressed HIV was assumed to be the major driving force, the virus cannot be eliminated in this model without intervention but only delimited. To boost CD4+ T-cell turnover during antiretroviral therapy (ART), when residual immune activation alone can no longer drive a significant flux, sequential waves of polyclonal T-cell proliferation and differentiation can be deliberately triggered using a variety of tested agents over a protracted period. ART will prevent infection of new cells. The model and the strategy are yet need to be tested in the nonhuman primate model of SIV infection.

## 13 Inflammation: SARS-CoV-2 infection as a case study

Inflammation is a unifying theme in basic and clinical research of SARS-CoV-2 infection, which underlies the ongoing COVID-19 pandemic. The lung is a major site of pathogenesis, and lung epithelial cells are major targets for infection. As suggested earlier, the aim of presumed immuno-neural computations in lung would be to strike a balance of cellular responses for the resolution of infection without overly detrimental inflammation.

The unique molecular patterns of viruses are initially detected by infected non-immune tissue cells, triggering the production of interferons and their intracellular products that participate in a variety of antiviral effects (46). In addition, they alert other non-immune tissue cells in the vicinity to the presence of the pathogen. They do it both directly, through cell-to-cell interaction, and indirectly, with resident and recruited immune cells acting as messengers and *via* sensory and reactive neuronal circuits. In parallel, infected cells alert the immune system, initiating immune responses locally and systemically. Preemptive responses are induced. For example, protective interferons are produced by uninfected cells. Growth and differentiation factors are also induced, to accelerate the orderly turnover of tissue cells, compensating for those being destroyed. This is a tissue response, as is the induction of angiogenesis upon hypoxia.

Several clinicians and other observers have considered or implied an exaggerated inflammatory response to infection of the lungs and other tissues to be a direct cause of the failure to clear the virus by the immune system in a subset of patients. Although it is difficult to discern cause and effect in the complex dynamics of failure, it is quite clear that timely clearance of the virus by well-coordinated response elements is a prerequisite to preventing immunopathology. Damage to the tissue inflicted by the virus itself and the associated stress likely destabilizes the interplay among these elements if not properly countered in time. Some attribute particular importance to diversion of systemic controls by the virus [evaluated, e.g., in (114) and (115)]. Accordingly, the virus interferes with the outward signaling functions of angiotensin-converting enzyme 2 (ACE2), its main receptor, creating imbalance between anti-inflammatory and pro-inflammatory signaling. Given evidence that tissue inflammation is orchestrated, locally and systemically, *via* local interactions within the tissue, it is simpler to adopt an infected tissue-centered view. According to this view, the major driver of dysregulated versus self-limiting inflammatory response is the persistence of infected cells in the affected organ and the attendant increase in functional stress. Naturally, impairment of systemic controls, associated with age, chronic inflammation, autoimmune disease, or microbiota changes in the gut can reduce the efficacy of local responses in several ways (116).

If tissue cells that are targeted by virus are relatively differentiated and mature – that is, if the progenitor cells serving as a tissue-specific source are not infected or are not the major target – then accelerating the flux may serve as a natural strategy to clear the virus by reducing its reproductive ratio below 1, and concomitantly to boost homeostasis in compensation for damage. Independently, the presence of virus specific T cells and of neutralizing antibodies likely reduces the virus replication competence in the local epithelium. The following observations regarding lung epithelial cells, the focus of interest, are consistent with this theoretical scenario, although detailed information is still missing:

### (a) Structure and variability of the flux

Uncovering differentiation hierarchies of epithelial cell types is still an active area of research [reviewed (117)]. There are nearly twice as many AT2 cells (alveolar type II epithelial cells) as AT1 (type I) cells, their progeny, but only a small subset (or subsets) of AT2 cells function as tissue stem cells in maintaining or repairing alveoli epithelium (117, 118). These cells undergo self-renewal sporadically during normal homeostasis and much more extensively after injury, accounting for large differences in their frequency among AT2 cells. (There are also phenotypic differences, but those are likely adaptively acquired, as discussed earlier, rather than representing the activation of distinct lineages or groups of tissue stem cells.) Concurrently, these proliferating cells serve as progenitor cells for new AT1 cells with several intermediate steps of differentiation and maturation (117). Knowledge about the intrinsic and external (cytokines, growth factors, extracellular vesicles) signals that drive normally quiescent AT2 progenitor cells to enter regenerative programs, and about the niche components that regulate and remodel these programs, is growing but incomplete (117, 118).

### (b) Accelerated turnover of AT2 during SARS-CoV-2 infection?

Several studies on COVID-19 deceased patients confirmed regeneration of damaged lung epithelium following infection, while others showed extensive alveolar damage and lung fibrosis (16). That such regeneration may in part at least be actively imposed early on as a virus washout mechanism is suggested by a study that compared asymptomatic cases to patients with symptoms and detected 7 analytes (IL-17C, MMP-10, FGF-19, FGF-21, FGF-23, CXCL5 and CCL23) that were higher in asymptomatic infection (119). These are known to be involved in tissue repair. If they were related to the control of symptoms, such control was apparently concomitant to efficient prevention of virus spreading and not a second phase reaction to damage. “It is possible that IL-17C, MMP-10, FGF-19, FGF-21, and FGF-23 act together to guarantee viral clearance and to promote lung tissue repair in asymptomatic individuals, and the early symptomatic individuals had a delay in this response” (119). Fibroblast growth factors recruit cells of the fibroblast family to alveolar stem cell niches, and those versatile and tunable cells (120–122) are critical for inducing the expansion of AT2 progenitor cells and their differentiation into AT1 (120). Moreover, there is evidence that angiotensin 1-7 (ang 1-7), a product of ACE2, can drive the proliferation and differentiation of AT2 progenitor/stem cells (123), and that the circulating levels of ang 1-7 are increased in severely ill COVID-19 patients (124) (they were not measured in others). The increase could be direct effect of SARS-CoV-2 or a consequence of inflammation.

### (c) Differential susceptibility to infection

In the upper airways, the basal cells serve as progenitor/stem cells. They are spared from infection and can proliferate and restore the damaged epithelium (125). In the gas exchange portion of the lung, the alveolar type II epithelial cell (AT2) is the main target cell type. It is the progenitor cell for type I epithelial cells (AT1), which cover 95% of the alveolar surface. Cell death and discordant immune response during infection contribute to alveolar damage and acute respiratory distress syndrome (ARDS). Only a small proportion of AT2 cells have detectable levels of ACE2 transcripts. AT1 cells express even less ACE2. The widespread damage to AT1 cells is likely due to the robust inflammatory response following infection as opposed to direct infection. Given those numbers, it is quite possible that the two subpopulations, the minority of AT2 cells that possess extensive self-renewal capacity (stemness) and the minority that are infectible, do not overlap; in that case, enhanced flux of activated progenitors into the general AT2 compartment can progressively dilute and decrease – rinse and replace – the infected fraction. Even if progenitors were as infectible as non-progenitor AT2 cells, it is quite possible that uninfected cells proliferate faster, therefore outcompeting the infected progenitors under a common feedback control. If theprogenitor cell population is unable to sustain infection for long, it is providing an essentially virus-free source for the alveolar epithelium.

### (d) Inherent longevity of infected cells

*In vitro* differentiated human nasal epithelial cells (NEC) are persistently infected with SARS-CoV-2 for up to 28 days post infection (126). These cells possess viral replication capacity despite the presence of an antiviral gene signature across all NEC cell types, but their persistent infection reflects limited cell death within the infected epithelium rather than replenishment of target cells and *de novo* infection. Persistent infection of epithelial cells by SARS-CoV-2 demonstrated in this *in vitro* model likely contributes to the spread of virus *in vivo* as well, both in the airways and in the lung. A virus evolves to evade the host cell antiviral machinery but also to avoid premature loss of its replication platform. Quite obviously, those infected cells that evade direct or indirect cytopathic effects of infection long enough to spread the virus in their vicinity are of primary concern. Active intervention by tissue-resident and infiltrating immune cells is required to reduce the efficacy of intercellular transmission and/or the time available for transmission, potentially resulting in viral clearance.

Together, these lines of evidence suggest that SARS-CoV-2 infection may boost the feedback-controlled flux through the AT2 and AT1 compartments, perhaps even independently of repair requirements.

**Figure 2:**
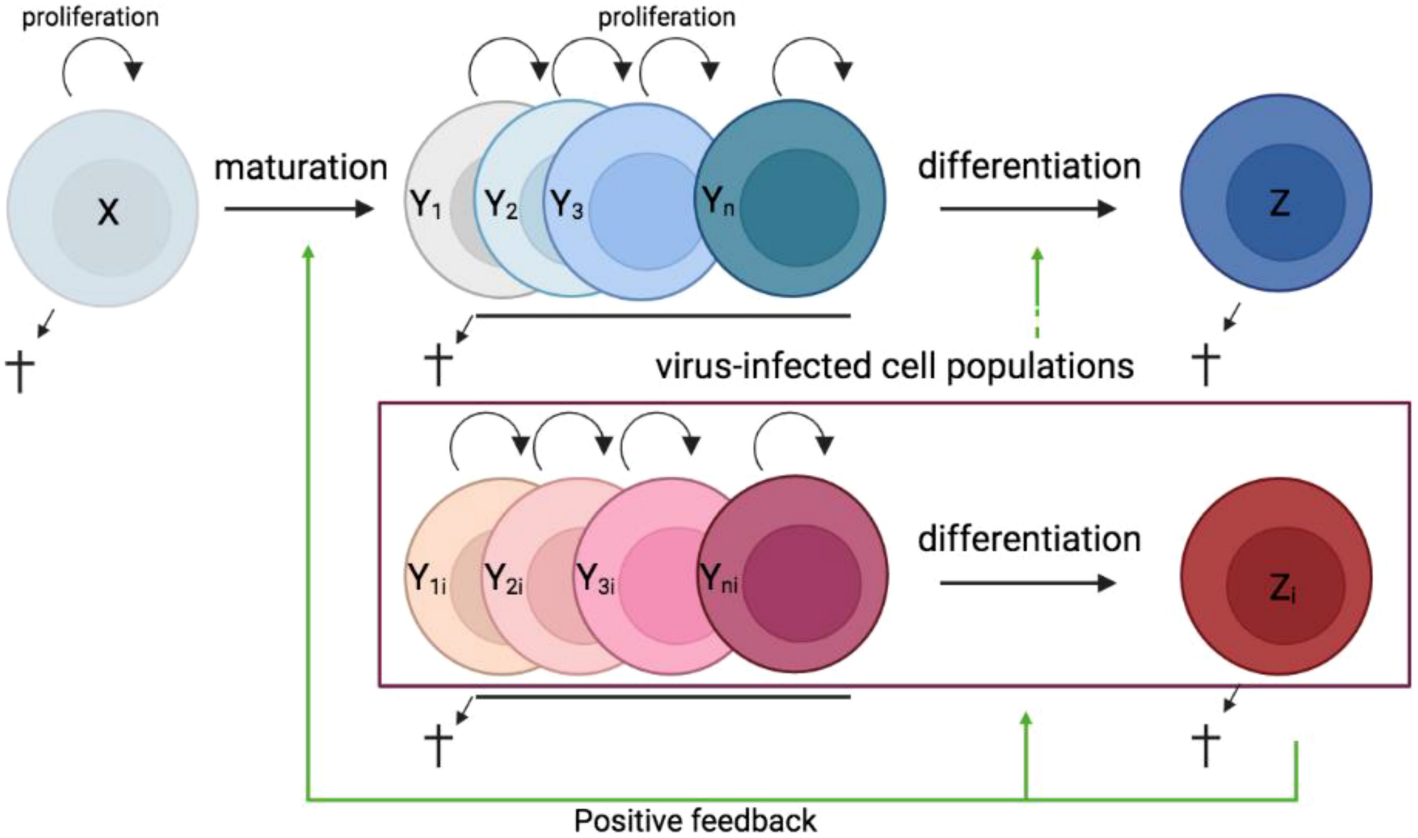
Schematic presentation of a simple feedback-controlled balance-of-growth-and-differentiation model considering lung alveolar epithelium infection with SARS-CoV-2. X, progenitor cells; Yk (k = 1, 2, …, n), AT2 cells in different maturation states; Yki, infected AT2 cells; Z, the subset of AT1 cells exerting feedback differentiation pressures; Zi, infected AT1 cells in that subset.

The model shown in **Figure 2** represents a minimal extension of the basic structure, **Figure 1**, required for its tentative application to the case of SARS-CoV-2 infection of the alveolar epithelium. It should be emphasized that this model is highly schematic and is not intended to reflect the actual biological complexity of the tissue. It suffices however to represent the overall structure of the flux and show the impact of variation at the source on the fates of the major cell types. This is the only purpose of the model. As by definition the compartment AT2 lumps together progenitor cells (tissue stem cells) and non-progenitor cells and also hides the maturation-differentiation structure of the latter, we introduce the minimal structure that is indicated by the literature into the mathematical representation. In the model, the main compartments are renamed, with X and Y referring to the progenitor and non-progenitor components of AT2; Z is AT1. We focus on active spread of virus in the AT2 cell compartments. Infection of progenitors (X) may probably be neglected, as reasoned in item *(c)* of the above-listed “observations”. We also assume that the AT1 cells (Z) do not actively spread the virus efficiently. The AT1 population is also presumably structured, with most but not all AT1 cells incorporated into a stationary thin interface above the underlining lung microvascular endothelium, forming a structure efficient for gas exchange. These cells are relatively isolated. We speculate that it is the role of free and relatively mobile differentiated AT1 cells that have failed to be incorporated to serve as a highly dynamic, short-lived, reservoir and as a dynamic measure of alveolar destruction/loss. Based on this speculation, the population Z, which in the archetypal model (**Figure 1**) mediates regulation of the overall balance of growth and differentiation, strictly represents this latter subset. Because cells in this subset are assumed to be terminally differentiated and short-lived, they would also be unable to efficiently spread the virus on their own. For simplicity, we finally assume that infected AT1 and AT2 cells are as functional as their uninfected counterparts. All these assumptions can be relaxed, but this is not necessary for our proof-of-principle purposes.

Given these observations and assumptions, it can be shown that boosting the flux of uninfected progenitors into the intermediate AT2 cell compartments can wash out all infected cells from the system within a short time. Boosting is most simply performed by increasing the normally minimal proliferation of progenitors. Sensing the presence of virus and signaling progenitors to proliferate is proposed to be mediated by immune cells, which steer the production of growth factors and of the ACE2 product ang 1-7. Increased flux from the progenitor end, in turn, is countered by a subsequent increase in the size of the differentiated, index population (a subset of AT1), accelerating epithelial cell differentiation *via* the postulated feedback loop. This shortens the transition time of infected and uninfected AT2 cells, reducing infection efficiency. The inherent longevity of infected cells demonstrated *in vitro* (along with differential susceptibility to infection, as discussed) suggests that shortening the average life span of AT2 cells could significantly curtail viral spread. The greater the flux, the faster the clearance of infected cells. These properties are generic, and would be applicable to many infective processes.

**Figure 3:**
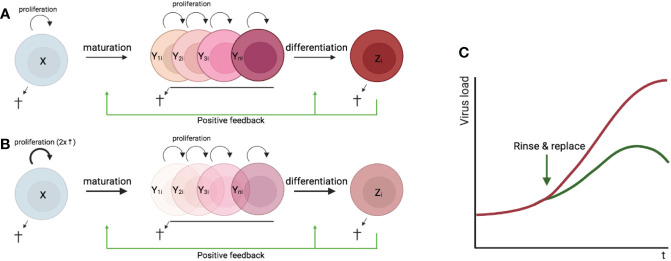
Schematic illustration of the effect of the “rinse and replace” strategy on SARS-CoV-2 infection. **(A)** Scheme illustrating the feedback-controlled balance-of-growth-and-differentiation model for virus infected lung epithelial cells. **(B)** Schematic illustration of the effect of a 2-fold increase of the proliferation rate of X cells. **(C)** The kinetics of virus load progression over time for baseline conditions and after a 2-fold increase of the proliferation rate of the progenitor cells. X, progenitor cells; Yki, (k = 1, 2, …, n), infected AT2 cells in different maturation stages; Zi, infected terminally differentiated cells belonging to the index subset of AT1 cells (see text).

**Figure 3** provides a concrete example. It shows the effect of increasing the progenitor proliferation rate on the course of infection, simulated using a mathematical model of the simplified infection process. The model is described in a mathematical Appendix (see **Supplementary Materials**).

In summary, it is suggested that rinse-and-replace may be operating in SARS-CoV-2 infection as a strategy alternative or complementary to the stereotypic response. In principle, a way to proceed, extending recent work (106), is to develop a full calibrated mathematical model in which antiviral innate and antigen-specific immune responses during mild-to-moderate symptoms infection also incorporate the hypothetical immune-mediated induction of accelerated epithelial regeneration. The need to recalibrate the model would instruct an elaborated search for prominent biomarkers and analytes that correlate with all these processes and together predict and impact the outcome. This could inform researchers on how to improve the vaccination program of choice and supplement it with adjuvants and immunomodulators aimed to enhance tissue preparedness.

## 14 Conclusion

The regulation of immunity and homeostasis is concomitant, flexible, and smart. It has been increasingly recognized that, to maintain the functional integrity of tissues, the body combines the information processing capacities of the brain with those that are possessed by the immune system. This requires that we selectively and cautiously apply to immunity cognitive science concepts and tools. Integrated neural and immune learning promote protection and resilience. The diversity of disease outcomes, such as asymptomatic *versus* symptomatic SARS-CoV-2 infection, mild disease *versus* severe one, as well as dependence of outcome on age and health status likely has a multifactorial basis. But it is conceivable that they partly arise from variances or discrepancies in the dynamic integration of information and its translation into an adaptive response, and not entirely from parameters of the direct confrontation of specialized effector cells with the spreading pathogen. Aberrant inflammation would accordingly represent a failure to adapt, resulting in uncontrolled expression of the molecular agents.

These perspectives redefine the limits of detailed bottom-up reconstructions, including mathematical modeling, of the molecular and cellular processes involved in the body’s response to infections and other insults. However, observing that the complex information processing often results in the selection of distinct classes of response and their combinations, and gaining insights into those, may help us in identifying biomarkers and their multivariate signatures that retrospectively correlate with preparedness for the challenge and in the design of therapies and vaccines. Effective immune-mediated response to acute infection by a pathogen such as SARS-CoV-2 may depend either on early immune elimination of the pathogen, before significant damage to inner tissues is caused by the pathogen and by the attendant inflammation, or if early elimination is not achievable – as when the host’s immune system is compromised – on engaging indirect, “smart” pathogen-elimination mechanisms associated with minimal peripheral damage. For example, when a stereotypic immune response to specifically eliminate the pathogen is not effectual enough, acceleration of the normal flux of proliferating and differentiating tissue cells may result in effective replacement of infected cells, and of damaged cells, by their uninfected precursors. This would suggests targeting homeostatic mechanisms in a tissue – *via* stimulation of endogenous tissue self-healing processes – along with the delivery of vaccines and adjuvants to imprint in advance patterns of inflammation concordant with a boosted turnover phenotype.

More generally, enhanced tissue-centered vaccination could be designed to imprint modifications of the composition and phenotypes of resident T cells and other immune cells and of neuro-immune circuits aimed to provide a more robust protection. Harnessing the full power of the immune system to prevent and ameliorate infectious disease requires advanced level of understanding of the immuno-homeostatic response. Although current biomedical, mathematical, and artificial-intelligence technologies efficiently generate and organize enormous amounts of insightful data, making the most of these advantages is still undermined by gaps in our conceptual understanding of the algorithms – the instructions for solving problems – installed by nature. Technological innovations and a continuing quest for organizing principles push the limits of biomedical research.

## Data availability statement

The original contributions presented in the study are included in the article/**Supplementary material**. Further inquiries can be directed to the corresponding authors.

## Author contributions

All coauthors contributed to the development of ideas. ZG wrote the first draft. ZG and GB formulated the illustrative mathematical model. GB performed simulations and calibration. All authors contributed to the article and approved the submitted version.
