# Supplementary material for: An integrative systems biology view of host-pathogen interactions: The regulation of immunity and homeostasis is concomitant, flexible, and smart
Source: Front Immunol. 2023 Jan 24;13:1061290. doi: 10.3389/fimmu.2022.1061290 (PMC9904014; doi:10.3389/fimmu.2022.1061290)
Supplement: Supplementary file 1 [file Presentation_1.pdf]

Supplementary Materials

Immune mediated boosting of lung epithelial cell turnover can restrain the spread of SARS-CoV-2

The mathematical model of lung epithelial cell turnover describes the population dynamics of progenitor cells *X(t)*, the AT2 cells in different maturation states $Y_k(t)$ $(k = 1, 2, \ldots, n)$ and the subset of AT1 cells exerting feedback differentiation pressures *Z(t)*. The turnover of the cells is determined by the feedback-controlled balance-of-growth-and-differentiation as shown in Figure 2. The respective homeostatic dynamics equations (virus-free case) are:

$$\frac{d}{dt}X(t) = \left(p_1 - d_1 \cdot Z(t)\right) \cdot X(t)$$

$$\frac{d}{dt}Y_1(t) = d_1 \cdot Z(t) \cdot X(t) + \left(p_2 - d_2 \cdot Z(t)\right) \cdot Y_1(t)$$

$$\frac{d}{dt}Y_2(t) = d_2 \cdot Z(t) \cdot Y_1(t) + \left(p_2 - d_2 \cdot Z(t)\right) \cdot Y_2(t)$$

$$\frac{d}{dt}Y_k(t) = d_2 \cdot Z(t) \cdot Y_{k-1}(t) + \left(p_2 - d_2 \cdot Z(t)\right) \cdot Y_k(t), \quad k = 3, \ldots, n$$

$$\frac{d}{dt}Z(t) = \left(d_2 \cdot Y_n(t) - D\right) \cdot Z(t)$$

Here the parameters p_1 and p_2 refer to the per capita rates of *X(t)* and $Y_k(t)$ subsets proliferation, respectively. The respective differentiation rate constants of *X(t)* and $Y_k(t)$ are represented by d_1 and d_2. The death rate of *Z(t)* cells is given by *D.* At steady state, we have

$$X_{stst} = \left(\frac{d_2}{d_1} - \frac{p_2}{p_1}\right) \cdot Y_1;$$

$$Y_{k-1,stst} = \cdot \left(1 - \frac{d_1 p_2}{d_2 p_1}\right) \cdot Y_{k,stst}, \quad k = 2, \ldots, n-1$$

$$Y_{n-1,stst} = \left(1 - \frac{d_1 p_2}{d_2 p_1}\right) \cdot \frac{D}{d_2};$$

$$Y_{n,stst} = \frac{D}{d_2}; \quad Z_{stst} = \frac{p_1}{d_1};$$

At steady state (Figure S1), *X* is much smaller than *Y* or *Z*, accounted by amplification of AT2, the Y_k cells, as they mature. Beyond this constraint, our choice of parameters is quite arbitrary, because, as explained, the correspondence of the compartments in our model with phenotypically identified populations is rather loose.

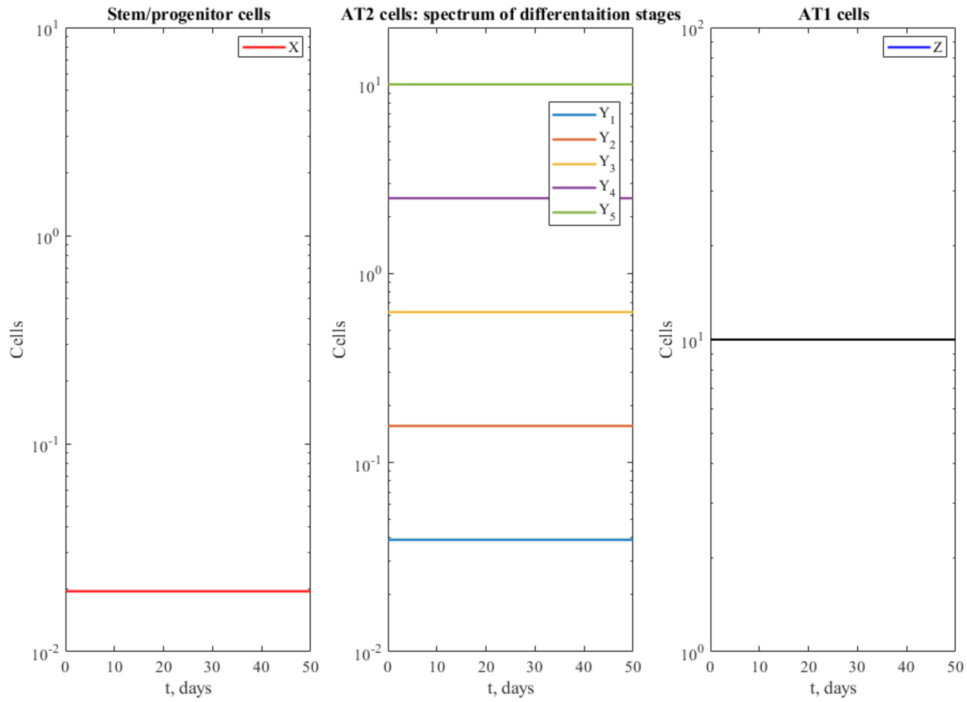

Figure S1. Illustration of homeostasis of the *X*, $Y_{k=1,\ldots,n}$, and *Z* subsets. The baseline parameters are: p_1=0.5, d_1=0.05, p_2=0.75, d_2=0.1, *D* =1.0. Time is in days, and other units are implicitly defined by the equations.

Note that ongoing damage to the structured AT1 layer would result in increasing *D*, as cells from the index/reservoir subset are recruited to fill the gaps and are destroyed in turn. These would activate a feedback response, resulting in an increased proportion of dividing *Y* cells, both progenitors and AT2, as observed (117).

When some epithelial cells become infected, the rate of spreading depends on the flux. Incorporating infected cells, Y_i and Z_i in the model's equations (below), and assuming for simplicity that all Y_i cells are infectible, the development of infection depends on the parameter p1. The development of infection is described by an extended version of the model which considers in addition the population dynamics of infected AT2 cell subsets, $Y_{ki}(t)$, *(k = 1, 2, …, n)*, and infected AT1 cells $Z_i(t)$, as follows:

$$\frac{d}{dt}X(t) = \left(p_1 - d_1 \cdot (Z(t) + Z_i(t))\right) \cdot X(t)$$

$$\frac{d}{dt}Y_1(t) = d_1 \cdot (Z(t) + Z_i(t)) \cdot X(t) + \left(p_2 - d_2 \cdot (Z(t) + Z_i(t))\right) \cdot Y_1(t) - r_2 Y_{1i}(t)\left(1 - \frac{Y_{1i}(t)}{Y_{1i}(t) + Y_1(t)}\right)$$

$$\frac{d}{dt}Y_{1i}(t) = r_2 Y_{1i}(t)\left(1 - \frac{Y_{1i}(t)}{Y_{1i}(t) + Y_1(t)}\right) + \left(p_{2i} - d_2 \cdot (Z(t) + Z_i(t))\right) \cdot Y_{1i}(t)$$

$$\frac{d}{dt}Y_2(t) = d_2 \cdot (Z(t) + Z_i(t)) \cdot Y_1(t) + \left(p_2 - d_2 \cdot (Z(t) + Z_i(t))\right) \cdot Y_2(t) - r_2 Y_{2i}(t)\left(1 - \frac{Y_{2i}(t)}{Y_{2i}(t) + Y_2(t)}\right)$$

$$\frac{d}{dt}Y_{2i}(t) = d_2 \cdot (Z(t) + Z_i(t)) \cdot Y_{1i}(t) + r_2 Y_{2i}(t)\left(1 - \frac{Y_{2i}(t)}{Y_{2i}(t) + Y_2(t)}\right) + \left(p_{2i} - d_2 \cdot (Z(t) + Z_i(t))\right) \cdot Y_{2i}(t)$$

$$\frac{d}{dt}Y_k(t) = d_2 \cdot (Z(t) + Z_i(t)) \cdot Y_{k-1}(t) + \left(p_2 - d_2 \cdot (Z(t) + Z_i(t))\right) \cdot Y_k(t) - r_2 Y_{ki}(t)\left(1 - \frac{Y_{ki}(t)}{Y_{ki}(t) + Y_k(t)}\right), \quad k = 3, \dots, n$$

$$\frac{d}{dt}Y_{ki}(t) = d_2 \cdot (Z(t) + Z_i(t)) \cdot Y_{(k-1)i}(t) + r_2 Y_{ki}(t)\left(1 - \frac{Y_{ki}(t)}{Y_{ki}(t) + Y_k(t)}\right) + \left(p_{2i} - d_2 \cdot (Z(t) + Z_i(t))\right) \cdot Y_{ki}(t)$$

$$\frac{d}{dt}Z(t) = d_2 \cdot Y_n(t) \cdot (Z(t) + Z_i(t)) - D \cdot Z(t)$$

$$\frac{d}{dt}Z_i(t) = d_2 \cdot Y_{ni}(t) \cdot (Z(t) + Z_i(t)) - D \cdot Z_i(t)$$

Here, r_1 and r_2 are the infection spreading parameters and p_{2i} are the per capita proliferation rates of infected cells. Our simulation demonstrates that a two-fold increase of p_1, which may indeed occur in response to SARS-CoV-2 infection (see text), can theoretically result in virus elimination after transient spreading (Figures S2 and S3).

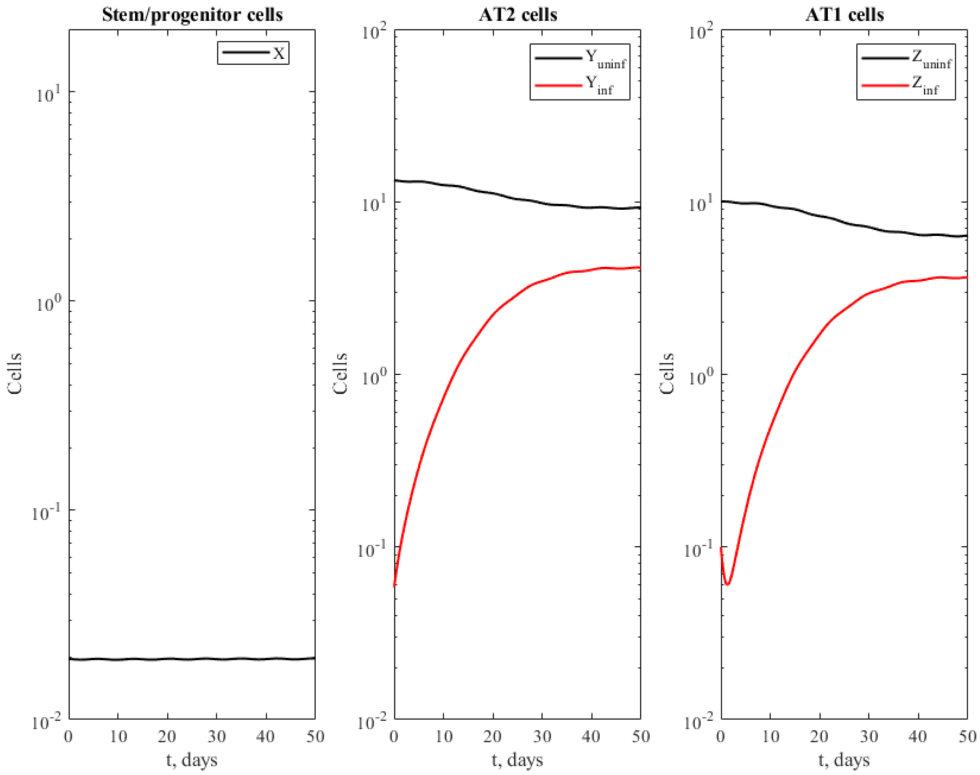

Figure S2. Development of infection with SARS-CoV-2. The baseline parameters are: p_1=0.5, d_1=0.05, p_2=0.75, d_2=0.1, D =1.0, n = 5; r_2 = 0.2; p_{2i} = 0.75. Fraction of initially infected is 1%.

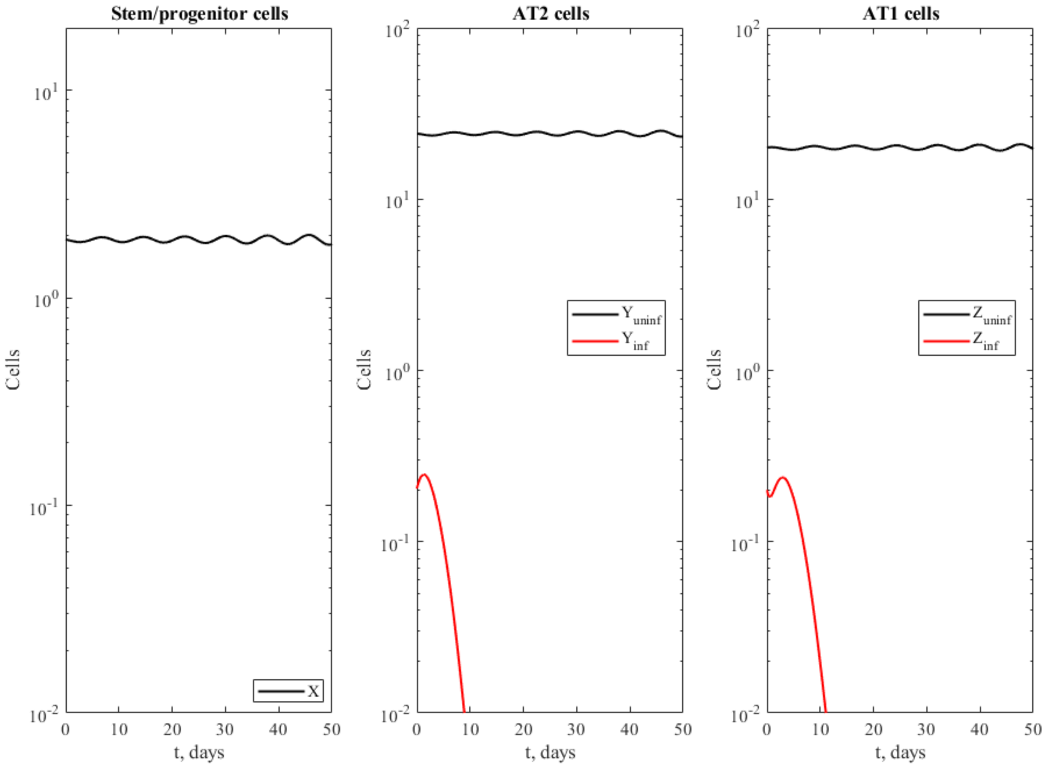

Figure S3. Extinction of infection with SARS-CoV-2 effected by increasing the proliferation rate of *X*-cells, p_1, by two-fold. The other parameters are unchanged.
